# Explaining the mixed findings of a randomised controlled trial of telehealth with centralised remote support for heart failure: multi-site qualitative study using the NASSS framework

**DOI:** 10.1186/s13063-020-04817-x

**Published:** 2020-10-27

**Authors:** Chrysanthi Papoutsi, Christine A’Court, Joseph Wherton, Sara Shaw, Trisha Greenhalgh

**Affiliations:** Nuffield Department of Primary Care Health Sciences, University of Oxford, Radcliffe Observatory Quarter, Woodstock Road, Oxford, OX2 6GG UK

**Keywords:** Heart failure, Complex intervention, Telehealth, Qualitative study, Socio-technical theory, NASSS framework

## Abstract

**Background:**

The SUPPORT-HF2 randomised controlled trial compared telehealth technology alone with the same technology combined with centralised remote support, in which a clinician responds promptly to biomarker changes. The intervention was implemented differently in different sites; no overall impact was found on the primary endpoint (proportion of patients on optimum treatment). We sought to explain the trial’s findings in a qualitative evaluation.

**Methods:**

Fifty-one people (25 patients, 3 carers, 18 clinicians, 4 additional researchers) were interviewed and observed in 7 UK trial sites in 2016–2018. We collected 110 pages of documents. The analysis was informed by the NASSS framework, a multi-level theoretical lens which considers non-adoption and abandonment of technologies by individuals and challenges to scale-up, spread and sustainability. In particular, we used NASSS to tease out why a ‘standardised’ socio-technical intervention played out differently in different sites.

**Results:**

Patients’ experiences of the technology were largely positive, though influenced by the nature and severity of their illness. In each trial site, existing services, staffing levels, technical capacity and previous telehealth experiences influenced how the complex intervention of ‘telehealth technology plus centralised specialist remote support’ was interpreted and the extent to which it was adopted and used to its full potential. In some sites, the intervention was quickly mobilised to fill significant gaps in service provision. In others, it was seen as usefully extending the existing care model for selected patients. Elsewhere, the new model was actively resisted and the technology little used. In one site, centralised provision of specialist advice aligned awkwardly with an existing community-based heart failure support service.

**Conclusions:**

Complex socio-technical interventions, even when implemented in a so-called standardised way with uniform inclusion and exclusion criteria, are inevitably implemented differently in different local settings because of how individual staff members interpret the technology and the trial protocol and because of the practical realities and path dependencies of local organisations. Site-specific iteration and embedding of a new technology-supported complex intervention may be required (in addition to co-design of the user interface) before such interventions are ready for testing in clinical trials.

**Trial registration:**

BMC ISRCTN Registry 86212709. Retrospectively registered on 5 September 2014

## Introduction

### Background

As the number of patients with heart failure grows and services are increasingly overstretched, telehealth is often depicted as a partial solution [1, 2]. A key determinant of outcome in heart failure is the proportion of patients on maximum tolerated therapy [3], but most heart failure patients are on sub-optimal doses of medication [4, 5]. The efficacy of telehealth solutions for heart failure has been widely studied in randomised controlled trials (RCTs), some but not all of which have shown a small benefit over usual care, as measured (for example) by reduced hospital admissions [1, 2]. Where benefit has been demonstrated, it has generally been attributed to more timely and detailed provision of biomarker data (e.g. blood pressure, weight, oxygen saturation), which allows prompt adjustment of medication in response to indicators of decompensation [2]. A new generation of trials has therefore focused on exploring and optimising the human component of the telehealth intervention [6, 7].

### SUPPORT-HF2: a randomised trial of a complex socio-technical intervention

SUPPORT-HF2 was a RCT in 7 UK sites, which aimed to improve the use of recommended medical therapy in heart failure as defined by evidence-based guidelines [7, 8]. A total of 202 participants (mean age 71; mean left ventricular ejection fraction 33%) were selected for being at high risk of adverse outcomes or high potential to benefit from remote management; they were randomised to ‘supported medical management’ (intervention) or ‘enhanced self-management’ (control, so named to avoid participants feeling they were in a no-treatment arm). Those in both arms submitted daily symptom reports and measurements of weight, blood pressure and heart rate, alongside free-text comments. In the intervention arm, home monitoring was combined with a clinical decision support system that provided risk rankings and tailored alerts. These were processed by the central clinical management (CCM) team—a cardiologist and heart failure specialist nurses (HFSNs)—who acted on these system-generated alerts to provide treatment recommendations (where relevant) to patients and their health professionals.

In the control arm, patients’ measurements were recorded in raw format only, without any processing by the clinical decision support system or the CCM team. Feedback messages were generated automatically as soon as patients entered their data. If readings fell outside pre-defined ranges (according to evidence-based guidelines), automated messages encouraged patients to contact their usual health professional. Participants in both arms were also provided with educational self-management modules on the tablet devices.

We describe the hypothesis, inclusion criteria, blinding and outcome measures for the SUPPORT-HF2 trial in the Appendix. More details can be found in previous publications, which describe the trial design and baseline participant characteristics [8] and the quantitative findings [7]. Despite being adequately powered, the trial showed no statistically significant difference between treatment arms in the primary outcome measure (proportion of patients on optimum medical treatment as measured by a ‘mean opportunity score’) or in various other endpoints including heart failure severity and disease-related quality of life [7].

Following the completion of the SUPPORT-HF2 trial, the technology is currently (February 2020) in development prior to commercial launch. The purpose of this paper is to describe a qualitative study into how the complex intervention of centralised remote support was interpreted by patients and staff and how, despite careful standardisation of the inclusion criteria, it played out differently in the different sites.

### The technology and linked service model

The patient-facing component of the technology consisted of a tablet personal computer, blood pressure and heart rate monitor, and weighing scales. The tablet portal listed current medication, health data entered by the patient, educational modules on heart failure and a messaging link to SUPPORT-HF2 clinicians. Patients were asked to complete a daily symptom checker (e.g. questions on activity and breathlessness). They then measured their blood pressure and weight (using peripheral devices supplied as part of the trial) and entered the data, which were transmitted from the peripheral devices to the SUPPORT-HF app on the participant’s tablet via Bluetooth. Patients could view their data in graphical form along with an indication of the normal range.

Data entered onto the tablet were sent securely to the trial CCM team by 4G (4th generation mobile network technology) or high-speed internet access (Wi-Fi) connection. Clinicians (including usual care teams) had access to patient data in raw format ‘with no ranking or interpretation’ via a secure login. For patients in the intervention arm, the CCM team was supported by a clinical decision support system which colour-coded patient data depending on the estimated risk of deterioration or deviation from globally defined parameters. The CCM team also drew on pre-established management plans, blood test results and other information from electronic records and compared these with optimal therapy targets via a central dashboard [8].

When a patient’s medication could be improved, the CCM team sent a letter to the patient’s GP. The lead study nurse would confirm that the advice had been acted on, either by receiving a message from the patient or during 3-monthly scheduled reviews. Patients in the control arm received automated messages encouraging them to see their GP if their measurements fell outside pre-defined ranges, but no treatment or drug titration recommendations were provided. Patients in both arms were advised that SUPPORT-HF2 was an ‘add-on’ service that did not replace standard GP or hospital care.

‘Usual care’ for heart failure in the UK generally consists of routine management in general practice and acute or elective admissions as needed to hospital in general medical, elderly care or cardiological wards. It also includes follow-up after a hospital admission by either a secondary care team (consultant or heart failure specialist nurse-led clinics) or in primary care (general practice, sometimes supported by community-based specialist nurses offering home-based or community clinic care). Sometimes, patients are discharged from an acute heart failure admission solely to the care provided by general practitioners, which can vary considerably depending on the latter’s skills and workload pressures.

### Research questions

This qualitative study took place alongside the SUPPORT-HF2 trial. The evaluation questions were as follows:

A. How and why was the intervention (and the control intervention) implemented differently in different settings?
B. What was the patient experience of the remote monitoring technology and service as delivered in the intervention and control arms of the trial?
C. What was the staff experience of the technology and service as delivered in the intervention and control arms of the trial?
D. What were the material, technical and clinical challenges in delivering the technology and linked service model as defined for the trial?

These questions were refined slightly as the study progressed, for example, to take account of the differences between sites in existing heart failure services, which had a bearing on how the intervention was embedded locally.

## Methods

### Study design, governance and ethical approval

We conducted a multi-site qualitative evaluation with data collection from all 7 sites. The study was overseen by the Studies in Co-creating Assisted Living Solutions (SCALS) [9] steering group which had a lay chair and representation from NHS, social care, external academics and patients (some of whom had heart disease). Ethical approval for the qualitative evaluation was obtained from the Oxfordshire South Central Research Ethics Committee (REC no. 15/SC/0553) in September 2015 and subsequent amendments (which covered all sites visited).

### Sampling and participants

Between May 2016 and September 2018, we interviewed and observed 51 people and collected various documents (summarised in Table 1).

**Table 1:** Summary of data sources

	Site A	Site B	Site C	Site D	Site E	Site F	Site G	Total
**SUPPORT-HF2 staff interviews**	11: consultant cardiologist/CI, trial manager, lead research nurse, 6 community HFSNs, bio-engineer, GP	3: consultant cardiologist/PI, 2 research nurses	4: research nurse, 3 HFSNs (1 hospital, 2 communities)	1: research practitioner	2: consultant cardiologist/PI, research administrator	1: hospital HFSN	1: consultant cardiologist/CI	23
**SUPPORT-HF2 patient interviews**	4 (including 1 spouse)		5 (including 1 spouse)	4	3	0	5	21
**SUPPORT-HF2 patient discussion group**		7 (6 patients, 1 spouse)						7
**Documents**	SUPPORT-HF2 study protocol Minutes of 5 meetings during the study set-up phase Minutes of a significant event review meeting (exploring the relationship between SUPPORT-HF2-driven drug up-titration and subsequent hospital admission) Approximately 50 email exchanges between site staff and researchers Approximately 10 emails between lead study nurse and researchers							Approx. 110 pages
**Total**	Directly involved in the trial: 25 patients, 3 spouses, 4 consultant cardiologists who were also local principal investigators, 10 heart failure specialist nurses, 4 research nurses, 1 trial manager, 1 bioengineer, 1 research practitioner, 1 research administrator							51 people

Patient participants included people living with heart failure who were taking part in the SUPPORT-HF2 trial (both arms), plus, where relevant, their family members or carers. They were initially approached by heart failure nurses and trial managers. We sampled to obtain variety in clinical background and different experiences using the technology. Interviews were semi-structured and lasted 30–90 min; most were conducted in patients’ homes. One patient discussion group (comprising 6 patients with heart failure and one carer) took place in a side room off a cardiology ward and lasted 90 min. Topic prompts included living with heart failure, experience of using the remote monitoring technology, and experience of clinical and research encounters.

Staff participants included consultant cardiologists, heart failure specialist nurses (HFSNs), general practitioners (GPs) and SUPPORT-HF2 trial staff (those managing the study from the central hub site and those recruiting and supporting patients in the 6 other sites). Discussions covered clinicians’ experiences of managing patients with heart failure, the role of technology in supporting patients’ self-management and communication with health professionals, and their experiences of being part of the SUPPORT-HF2 trial.

Documents included national and international guidelines for acute and chronic heart failure management, the annual output from the National Heart Failure Audit, heart failure nurse operational procedures and standard clinical information collection templates for patients outside the SUPPORT-HF2 trial.

### Data management and analysis

Forty-two of the 51 interviews were audio-recorded with consent and transcribed; the remainder were recorded as contemporaneous notes. Anonymised transcripts and other data sources were imported into NVivo 12. We analysed qualitative data thematically and produced summary narratives that we progressively refined over time, adding each new data item to an increasingly nuanced account of the overall patient, carer and staff experience [10]. We discussed ongoing data collection and emerging findings in regular team meetings that helped us shape interim analysis and further data collection.

### Theoretical framework

We drew on socio-technical theory—of which there are many interpretations and versions [9, 11]. Broadly speaking, socio-technical theories depict technologies as part of complex systems; they focus on how those technologies are perceived, interpreted and used by individuals and how the use (or non-use) of particular technologies affects and is affected by the wider system. A socio-technical approach to technologies in the home considers how and to what extent they are ‘domesticated’, both technically (e.g. do they work; are they dependable?) and symbolically (e.g. are they reassuring or threatening?) [12]. A socio-technical approach to technologies in the workplace considers how they influence work practices and routines and how their use either enhances or challenges professional standards of quality, safety and equity of care [11, 13]. An early and important contribution to socio-technical theory was Albert Cherns’ work on the need for extensive reconfiguration of work practices to ensure that new technologies are smoothly embedded in processes and systems [13].

**Fig. 1:**
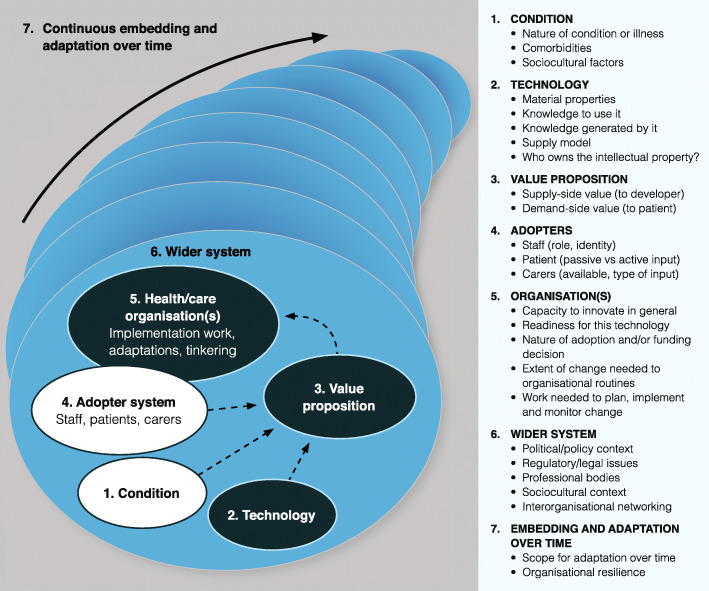
The NASSS framework for studying non-adoption and abandonment of technologies by individuals and the challenges to scale-up, spread and sustainability of such technologies in health and care organisations (adapted from Greenhalgh et al. [14])

The specific socio-technical lens used for this analysis was NASSS, a multi-level theoretical framework which considers non-adoption and abandonment of technologies by users and challenges to organisational scale-up, spread and sustainability (Fig. 1) [14]. NASSS has 7 domains, each of which may be characterised by complexity (that is, unpredictability, interdependence with other domains and unintended consequences): the illness or condition, the material features and functions of the technology, the value proposition (both financial and non-financial value, including negative value, associated with the technology), the intended users (staff, patients and carers), organisational characteristics (including general capacity to innovate, readiness for the technology, extent of change needed to revise existing routines and the work needed to plan, implement and monitor the change), wider system issues (including the technical context, such as availability of broadband, and the policy context, such as how national and regional policy decisions are playing out locally) and how all these domains interact and evolve over time. We analysed each domain (and their interactions) to consider how SUPPORT-HF2’s ‘standardised’ socio-technical intervention played out differently in different trial sites. The application of NASSS to a process evaluation is an example of what Mills et al. call a ‘type 4 logic model’—that is, a systematic way of exploring interdependencies in dynamic systems [15].

## Results

The study generated a rich and heterogeneous qualitative dataset comprising several hundred pages of interviews, ethnographic field notes, email exchanges and extracts from documents. Not all of these data were pertinent to this analysis; the NASSS framework guided us to relevant material. Below, we draw on the domains of the NASSS framework (Fig. 1) to explain how the trial was received and operationalised in the different settings, leading to different impacts on the adoption process and the care pathway.

### Domain 1: The illness

Heart failure is a heterogeneous condition with a wide range of severity and different underlying causes and comorbidities; because of its links to smoking and body mass index, it disproportionately affects the poor and those with low health literacy [1]. Patient participants in the SUPPORT-HF trial had a mean age of 71 (standard deviation 11) years. A high proportion had comorbidities (the commonest, in order of frequency, were atrial fibrillation, hypertension, ischaemic heart disease, diabetes, chronic kidney disease, chronic obstructive pulmonary disease and past stroke). The mean ejection fraction was 37% (SD 12%); 66% had heart failure with reduced ejection fraction. Their heart failure varied in severity from class 1 to class 4 on the New York Heart Association scale. All researchers were blinded to which arm the patients were in when they were assessed, so the data below reflect the experiences of patients in both intervention and control arms. As noted above, patients in both arms received the same equipment, though their health providers were involved in different ways.

The patients we interviewed (sometimes together with family members) had different experiences living with heart failure and other medical conditions; came from different socio-economic backgrounds, from rural and urban settings; and had varied views on the technology.

Heart failure patients typically described a range of symptoms alongside several comorbidities:

Had been generally suffering with breathlessness and tiredness, and pains in my chest, etc. Being a typical bloke, I did not really do anything about it. [Then] I fell down the stairs, top to bottom. And fractured a vertebrae. […] they have introduced and taken away [medicines] I’m quite light-headed most of the time. But I got kind of a double whammy on that, because I’m suffering from depression and anxiety as well […] I get migraines quite badly. I’ve got osteoporosis in the spine, as well […] memory’s not very clever either. Interview 9, heart failure patient, site E

Another patient described her effort to adjust to her new way of life, which required changes in the things she enjoyed and meant she had to rely on extra support:

And if I get up very slowly I’m alright, as the day goes on, I get better. I have got support rails. And I take a stick. But I try to get round the garden, to get a bit of exercise. Of course I cannot take [the dog] out now […] I do like to keep up standards and things as much as possible. I do have help in the house. Because some of the housework was rather difficult. Interview 7, heart failure patient, Site G

In some cases, the way patients experienced their conditions was clearly linked to how they engaged with the SUPPORT-HF technologies. Many trial participants suggested that the technologies captured well some of the key indicators for their heart condition. Others, however, found it more challenging to use the technology in the wider context of their health difficulties. A man in his early 50s with heart failure became withdrawn and abandoned the technologies when he unexpectedly found out he was not eligible for a heart transplant following a 6-year wait, due to a comorbid condition. Other patients with severe heart failure and/or comorbidities found the intervention extremely burdensome; some felt physically unable to use the weighing scales (one was an amputee; another had difficulty with balance). One man with severe fluid retention awaiting a heart transplant could not bear the anxiety engendered by a data trend indicating weight increase. Another lady explained how she was initially frightened by the diagnosis and felt the technologies made her even more anxious, but this subsided as she became more comfortable managing her condition. Others viewed the technology primary purpose as supporting their immediate health concerns (e.g. one believed, incorrectly, that stable weight indicated that a different comorbid condition was under control) and placed less value on monitoring stable indicators.

The staff gave examples of patients who, for various reasons including comorbidity, anxiety or wider life issues, declined to participate in the trial, withdrew after enrolment or expressed relief when the trial ended. Such cases were relatively rare, though withdrawal rates varied across sites.

Whilst the specific nature of the participants’ illness had a major influence on whether and to what extent they accepted and used the technology, because of the randomisation process, these differences were evenly distributed between the intervention and control arms of the trial. It is worth noting that of 363 patients assessed as eligible for the trial, 126 (35%) were considered ‘unsuitable’ by clinical staff who were participating in recruitment. Reasons for this are discussed in other sections below.

### Domain 2: The technology

The tablet device used for SUPPORT-HF2 had been co-designed with patient input [16] and tested in a usability study (without the central support component) in which patients suggested some minor adjustments [17, 18] before the trial began. In an early qualitative study, patients were visited at home to document the variable and emergent ways in which they appropriated the technology, made sense of it and embedded it in their routines [18]. Given this careful preliminary design phase, it is reassuring that most patient participants and their carers described the SUPPORT-HF2 technology as ‘easy to use’.

But this iPad is quite simple. You just switch it on. And it - You pick what you want to do. Weighing, or blood pressure. Just touch it, put it on there. Put the thing on, it’ll do it. The iPad will pick up the thing. It’d show you what it had read. Boom. Thank you for doing it. Done, that’s it - boom, finished. That was it. Interview 8, heart failure patient, Site G

The technology was not, however, ‘plug and play’. It was necessary to visit every participant at home, usually on two occasions, to set up the equipment and show the patient how to use it. This was a time-consuming process (typically 2 h for the first visit and 1 h for the second), though it is not clear how much of this time was spent setting up the equipment and how much on requirements specific to the clinical trial. Some patients needed extra support to feel confident with it.

Patient participants raised a number of practical issues with the home equipment. Sometimes, synchronisation of readings via Bluetooth between the monitor and the tablet was slow or there were problems with data transmission due to fluctuations in 4G signal; these problems usually resolved if a Wi-Fi connection could be established. These transmission issues sometimes led to patient confusion as they could see that the data had not been ‘sent’. Some felt they could not rely on the machine and assumed a responsibility for confirming their data had been received by the central support team and their medicines were listed correctly.

When technical difficulties occurred (quite commonly with WiFi or Bluetooth connection, but also due to malfunctioning equipment), some patients found solutions themselves, but in most cases, input from the trial team and local nurses was crucial for resolving them quickly. Research nurses used a workaround to help manage synchronisation or transmission issues. They gave participants a separate phone number to use if the messaging facility of the tablet was not working, and if they had not received any data or messages from a participant for a day or two, they would phone them to check that all was well. In most cases, such problems were due to patients not connecting properly to their own Wi-Fi.

Occasionally, the ‘easy-to-use’ tablet malfunctioned because of user error.

Another person had somehow changed the setting so it was now in a foreign language. And we couldn’t read it to correct it. Had to get [CCM research nurse] involved and she talked us through screen by screen. Research administrator, Site E [SUPPStaff15]

The trial design allowed for some iterative changes in the technology. Most of these were made to facilitate data collection for research, though the bioengineer’s input was sought to make adjustments to the dashboard with the aim of improving the clinical care of participants.

### Domain 3: The value proposition

The SUPPORT-HF technology was part of a research trial, so (during the study period at least), there was no immediate requirement to demonstrate affordability or value for money, and the varying technology budgets of the different sites did not affect adoption decisions. It is worth noting, however, that the software changes described in the previous section were resource-intensive and costly, and this seems to have constrained the number of co-designed modifications. In the case of one patient, for example, the system did not flag up a clinically important weight increase because it did not occur rapidly enough (i.e. over a 3-day period—the conventional assessment time frame). The central support team worked with IT colleagues to adjust the system to change the alert algorithm in this case, but such changes were labour-intensive and hence expensive and unscalable. The CCM lead nurse commented that further refinement of the dashboard, enabling safety alert flags to be easily individualised, could improve the value of the technology since it would allow ‘personalisation’ of the alerts.

With any technology, there is a trade-off between advanced functionality and development cost. A basic product with ‘must-have’ but not ‘nice-to-have’ functions may prove more cost-effective. The SUPPORT-HF2 blood pressure monitors did not, for example, include the now widely available ‘Irregular Heart Beat Indicators’ (based on variability in the R-R interval on ECG). Since decompensation in heart failure is often triggered by new-onset atrial fibrillation, community staff suggested this might have been a useful inclusion, although potentially pushing up the price. The absence of rhythm detection meant that the possibility of undiagnosed atrial fibrillation affected confidence in remotely recorded blood pressure readings.

### Domain 4: The intended adopters

Aside from issues raised by the nature of their health conditions (domain 1), most patient participants were interested to see their readings and described the technology as well-designed. They used the tablet and the peripheral devices without too much difficulty and saw great value in monitoring their condition, especially in terms of gaining reassurance and legitimising help-seeking when they needed clinical care. Those who continued to use the tablet until the end of their study participation regretted having to hand it back at the end of the trial. Others, however, described it as ‘intrusive’ and found it impossible to get into the routine of regular monitoring; a few were sceptical about its role in supporting clinical care:

Somebody is looking at a screen, without seeing the patient, saying what you should do. Without actually examining the patient. So, that was a bit - That wasn’t really - Yeah, I was [skeptical], about that. Yeah. That they don’t actually see the patient, they’re just based on figures. Interview 13, heart failure patient and wife, Site C

The trial also assumed that patient users would largely remain static in their homes where they would have good access to the devices on an everyday basis. This was not the case for more active patients (especially those who travelled away from home) who found this too restrictive, and one or two withdrew from the study for this reason.

Despite some challenges with implementing the intervention (see domain 2), most staff also expressed positive views on telehealth technology enabling remote support in heart failure care (a finding that should be interpreted in the context that these staff interviewees were almost all linked to the study in some way so had ‘skin in the game’). Many HFSNs felt that patients had benefited from both the technology and the day-to-day input from the central support team.

There were, however, tensions in the way different professional groups made sense of the potential of the technology in heart failure care. For example, some consultants thought that the reluctance of HFSNs to engage with the technology stemmed from a belief that the technology would replace their jobs. The nurses themselves had a more nuanced view. They expressed concern that if routine uptitration in relatively stable patients was carried out by a telehealth service, they would lose the valued reference point of the straightforward, treatment-responsive patient and be left with a case load of unrelentingly complex and unstable cases.

Another concern of nurses in particular (and also expressed by some patients) was that the structured and algorithmic element of the telehealth intervention would miss important aspects of quality care. Technology, HFSNs felt, provided narrow and decontextualised information and could not replace the nuanced and holistic assessments that experienced clinicians undertook on their patients—including, for example, home visits which gave them rich information about the patient’s environment and allowed them to observe how the patients approached activities of daily living and medication management. The cardiologist chief investigator, however, rejected the nurses’ characterisation of the SUPPORT-HF2 intervention as crudely algorithmic. On the contrary, he argued the intervention design recognised and accommodated the need for human input and judgement where necessary, whilst attempting to streamline redundancy and repetition.

The question of professional lines of responsibility for patients, especially in terms of nursing care, also caused concern amongst clinical participants. The SUPPORT-HF study team envisaged that the intervention would run alongside and complement usual care and not challenge the work of local heart failure teams, and the trial protocol assumed that data generated by the decision support dashboard and the advice given by the CCM would be uncontested and unproblematic. In reality, clinical disagreements sometimes led to conflicts between nurses from different teams and required time-consuming efforts to resolve differences and negotiate professional boundaries:

What we have all found is that co-managing doesn’t work. Because the numbers are saying one thing but we have visually seen the patients and we may have known those patients for a long time, know their complexities and when the SUPPORT-HF team give advice we sometimes disagree with some of the decisions. We’ve had to unpick that, it’s very complex. It meant a lot of sitting in a back room making calls Community HFSN, Site A [FACEHFSN3]

These conflicts appeared to stem from two things: an implicit knowledge hierarchy in which the technology’s decision support system appeared to override their personal knowledge of the patient and the fact that patients in the control arm were sent a generic alert (and a prompt to see their usual clinician) but no specific support. Neither the local trial staff nor the non-trial clinicians knew which arm the patients had been randomised to, so there was sometimes uncertainty about who should take action when an alert occurred.

Principal investigators in the study sites (who were all cardiologists) did not perceive role conflicts with the central remote support team, with the one exception of the cardiologist in site B, who vetted that team’s recommendations. He said he knew the patients personally and could take account of exceptions, and he felt the GPs (whose views on telehealth had been influenced by adverse past experiences) would have greater confidence in implementing recommendations from him than from the CCM.

### Domain 5: The organisation

Study sites joined the trial with different existing service models, staffing levels, technical and clinical capabilities, and distribution of professional responsibilities. They had different local demographics, histories, cultures and past experiences with telehealth and other technology projects. Whilst recruitment criteria for SUPPORT-HF2 were the same in all sites, the organisational service context for each site (Table 2) led to variability in how the intervention was implemented and the extent to which it aligned or clashed with existing organisational and inter-organisational routines.

**Table 2:** Cross-site comparison – contexts and implementation

	Site A: large city, South East England	Site B: major city, Northern Ireland	Site C: major city, East Midlands	Site D: rural, South West England	Site E: major city, North West England	Site F: urban area, South East England	Site G: major town, South England
**Existing service prior to SUPPORT-HF2 trial**	Consultant-led clinics and inpatient management Standard primary care diagnosis and ongoing management						
	Hospital HFSN team, community HFSN teams serving HFrEF only	Hospital and community HFSN teams	Hospital and community HFSN teams	Hospital and community HFSN teams	Community HFSN team in one part of CCG only	Hospital and community HFSN teams	No community HFSN team
**Staff involved in SUPPORT-HF2 trial**	Chief investigator, secondary care HF nurses, SUPPORT HF trial team	Local PI, 2 research nurses	Local PI, secondary care-based lead HFSN and 2 communities	Local PI, secondary care HFSNs, 2 research practitioners	Local PI, research administrator	Local PI, secondary care-based HFSN	Local PI
**Setting for recruitment**	Study lead nurse recruited from wards or clinics, community HF nurses in the early phase only	Research nurse recruited from CCU and other wards or by letters sent out post-discharge	Recruited by lead HFSN then later by research nurse and community HFSNs from the clinic	2 research practitioners, secondary care HFSNs and local PI recruited from wards and hospital clinics	By local PI in hospital clinic	By secondary care nurse in hospital clinic	By local PI in hospital clinic
**Clinical profile of participants in this site**	As per protocol, no specific distinction described.	As per protocol, no specific distinction described.	Patients were recruited only when ready for discharge from the hospital clinic or HFSN service.	As per protocol, no specific distinction described.	Patients were targeted if they were particularly unwell and deemed in need of monitoring between 6 monthly clinic visits.	In time, with appreciation of RCT design, staff avoided recruitment of any patients deemed too unstable for the control arm.	As per protocol, no specific distinction described.
**How technology use by patients was supported during the trial**	By CCM team	By local research nurse, supported by the CCM team	By local research nurse, supported by the CCM team	By local research nurse, supported by the CCM team	By CCM team	By CCM team	By CCM team
**Extent to which the model was integrated into clinical pathways**	Initially integrated, later separated due to role overlap	Limited: resistance from both hospital and community HFSNs and GPs	Partial: intention was for participants to be looked after by the CCM team, but sometimes, the hospital HFSN accessed patient data.	Partial: integrated with secondary care only as community HFSN service was resistant	Not integrated: following recruitment, participants were looked after by the CCM team.	Not integrated: following recruitment, participants were looked after by the CCM team.	Not integrated: following recruitment, participants were looked after by the CCM team.

In site A (the main ‘hub’), some of the patients initially recruited by the CCM lead research nurse were also receiving support from a nurse-led community heart failure service. It proved very difficult to align the trial protocol with the existing community care routine, so the clinical teams eventually arrived at a policy of keeping trial participants separate from the community-based service and recruiting patients from areas with less intensive community-based services. Site D also had a nurse-led community heart failure service; these HFSNs appeared less interested in the trial and made few referrals (in a few cases, they actively resisted cooperation). But, the hospital-based team in site D actively recruited patients to SUPPORT-HF, partly because they saw remote monitoring as a solution to long geographical distances.

In site B, neither community HFSNs nor GPs recruited patients or participated actively in the study (probably because of a negative experience with a previous telehealth trial); the hospital research nurse and principal site investigator (a consultant cardiologist) took on responsibility for recruitment and prescribing. In site F, hospital-based HFSNs had a large number of complex or unstable patients which they deemed unsuitable for the trial (given the lack of active management in the control arm) and decided to refer only the more straightforward patients whom they would normally have referred to community HFSNs. In this site, the trial served as an alternative rather than an add-on to the usual care pathway, which may also explain why recruitment stalled part way through the trial.

In site C, there was no local cardiologist involvement in the trial; the community HFSN team actively recruited patients whom they were about to discharge to their GPs for follow-up, because they saw the intervention as a useful supplement to existing care. Although these community HFSNs would sometimes continue to see patients who were participants in the trial, no interprofessional conflicts were described with the CCM team (unlike in site A). In sites E and G, there was no specialist community nursing to augment GP management of heart failure; SUPPORT-HF2 was welcomed by local clinicians as it aligned well with pressing service needs. In site E, the principal site investigator (consultant cardiologist) deliberately sought to recruit patients in localities with substantial pressures on hospital outpatient appointments and lack of community HFSNs. In site G, the hospital outpatient clinic seized the opportunity to ‘hand patients over’ to the trial due to the lack of resources for specialist care post-discharge.

It fits in really nicely with the existing infrastructure. We need a way of surveilling all those being up-titrated. And to monitor the sicker ones such as those receiving home IV diuretics. So we can use it for disease management but also to see progression, and see when they’re falling off their perch. Consultant, PI, Site E [SUPPStaff11]

This is consistent with the SUPPORT-HF2 protocol, which hypothesised the value of digital health interventions to be higher ‘in contexts where quality of care is (on average) suboptimal with substantial unwarranted variability at the provider-level.’ ([8], p.62). The different experiences across sites, however, highlight the challenges of running an RCT alongside existing standard care that may be historically embedded and involve staff with varying degrees of buy-in to the research.

**Fig. 2:**
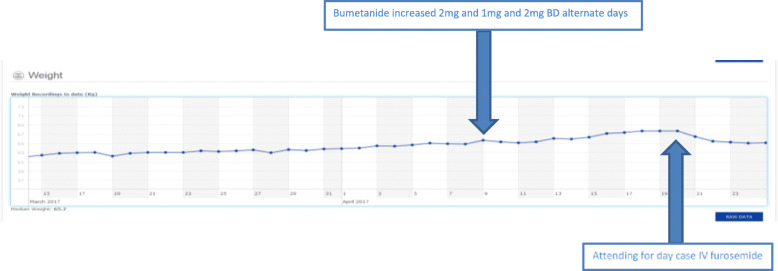
Longitudinal weight readings entered by patient and active interventions recommended by central support staff based on the SUPPORT-HF algorithm

The different existing routines and practices (and the extent to which front-line staff were able and willing to adjust them) had a direct impact on the care pathway in the SUPPORT-HF2 trial. The rationale behind the trial included the importance of a tight feedback loop in deteriorating patients. This was sometimes achieved successfully, with local input from site staff and active intervention by the central support unit via the SUPPORT-HF2 dashboard (see Fig. 2).

Sometimes, however, the feedback loop did not work as intended, mainly due to challenges in communicating recommendations to GPs, establishing whether these had been actioned (either by GPs or by patients themselves) and confirming time frames for up-titration. Factors outside the control of the trial staff and local clinicians (for example, the unavoidable use of traditional ‘snail mail’ letters with GPs who at the time used neither email nor faxes) caused considerable delays in some sites.

### Domain 6: The wider system

This telehealth trial tested the effectiveness of remote monitoring with centralised clinical support for heart failure patients but also sought to collect a dataset for machine learning and algorithm training to improve early warning prediction of deterioration. This was aligned with a wider orientation for automated, data-driven clinical care in the UK, supported by policy priorities for the development of artificial intelligence-enabled solutions [19, 20]. Despite being forward-looking (envisioning a future in which heart failure clinicians’ work would be supported and made more efficient by state-of-the-art technologies, thereby potentially overcoming workforce shortages and funding shortfalls), the trial encountered practical challenges as it relied on legacy infrastructures for data transmission and collection. For example, in site B, half of all potentially eligible patients could not be randomised due to the lack of broadband coverage in the area where they lived. The study also aimed to collect data on clinical investigations, events and medications directly from participants’ electronic health records to complete its dataset. However, in the majority of sites, it was not possible to achieve this level of integration with secondary and primary care systems in the duration of the trial. Instead, the trial team attempted to collect this information directly from patients, their clinicians or local research staff.

### Domain 7: Evolution over time

There were three key areas where evolution over time affected the way the trial was operationalised. Firstly, over time, there were substantial changes in both staffing and priorities, which meant that the interpretation of the trial aims and the way it was embedded in the service changed as well, and this was reflected (for example) in fluctuating recruitment rates. Secondly, heart failure patients with complex comorbidities received care from multiple health professionals, and their health status changed during the trial, which required constant updating of patients’ medication and other clinical details on the SUPPORT-HF2 dashboard in the absence of data integration (updates were planned every 3 months for the control group and every 2 weeks for the intervention group). In the face of emergent challenges, the CCM nurse demonstrated significant adaptive capability, resolving technical difficulties and co-ordinating with patients and other health professionals to try and close the feedback loop. Thirdly, as mentioned earlier, adapting the technology to the level required for emerging trial needs proved impossible given funding restrictions.

## Discussion

### Summary of main findings

This qualitative study of the SUPPORT-HF2 trial produced a number of key findings. First, heart failure patients and clinical staff saw potential in a technology that supports regular data monitoring but also identified challenges in mainstreaming its use. Trial participants experienced the technology as usable and useful; they were (mostly but not invariably) reassured by the monitoring and support and found that the data informed and legitimised their help-seeking behaviour. However, sicker patients and those with comorbidities found regular monitoring burdensome (something we have demonstrated previously in relation to self-monitoring more generally [12]) and described feelings of alienation or saw the technology as a constant reminder of their ill health. Clinical staff valued the technology, but they also identified challenges in reconciling what they saw as an algorithmically driven intervention with holistic, personalised care based on long knowledge of the patient. Co-management of patients by both the central trial team and community HFSNs produced tensions of professional responsibility and judgement.

The second key finding was that, as predicted by the NASSS framework, significant human effort was required to embed the intervention and make it ‘work’. It was often necessary for patients or staff to resolve issues with network or device connectivity to ensure that data were collected and transmitted reliably. Patients felt responsible for confirming that their data had been transmitted and providing context for them. Clinical staff had to engage in significant co-ordination work to communicate recommendations to other health professionals and/or to patients, establish whether these recommendations had been acted on, and confirm steps and time frames for medication changes. In contrast to the assumptions in the protocol, these were not neutral information exchange tasks but required professional negotiations and careful management of boundaries and expertise between clinicians.

The third key finding was that, again as predicted by the NASSS framework [14], implementation of this complex trial was highly variable across sites because of local contextual issues (although inclusion criteria were standardised). Existing service models, staffing levels and distribution of responsibilities, capacity at each site and previous experience with telehealth all had a strong influence on the extent to which the intervention was accepted and implemented. In some settings, technology-supported remote specialist input was seen as potentially useful in supplementing existing care and readily mobilised to fill specific gaps in service provision (including, in one site, using it as a safety net for sicker patients). Sites with relatively comprehensive existing services accommodated the SUPPORT-HF2 trial mainly by targeting it to patients considered stable enough not to need local HFSN input. In some sites, the trial was actively resisted for historical reasons. Recruitment and implementation were also influenced by concerns that patient care should not be compromised if they were allocated to the control arm.

### Comparison with other studies

The use of telehealth in heart failure management remains controversial, with some clinicians and policymakers strongly enthusiastic [21–24] and others unconvinced or opposed [25–27]. In a recent review carried out by our team, we found that telehealth showed more benefits when ‘usual’ heart failure care was sub-optimal and when it targeted high-risk patients—its success improved with the number of variables monitored, the frequency of monitoring and the timeliness of human remote support [1]. However, the literature on telehealth in heart failure (especially trials) appeared to suffer from publication bias, and poor recruitment was a common challenge [1, 28].

Factors shown to account for poor uptake of telehealth by heart failure patients include preference for face-to-face encounters, lack of perceived relative advantage of the technology over existing care, physical or mental impairments and lack of confidence that limit patients’ ability to use the technology [1]. Consistent with our findings on the implementation of SUPPORT-HF2, several studies have also identified clinician non-acceptance as a major factor in the low uptake of telehealth [29–33]. It has been proposed that champions in telehealth service development could support acceptance as they work to enthusiastically cultivate relationships and promote and legitimise telehealth [34]. Technical factors identified in this study, such as challenges with bandwidth availability (esp. in rural areas) and connectivity, and lack of interoperability with electronic patient records, have also been identified elsewhere [1].

Previous research has consisted of either outcome-focused RCTs or (less commonly) qualitative studies in a non-RCT setting. This study has extended the knowledge base by producing ‘behind-the-scenes’ qualitative insights into the socio-technical challenges of running a multi-site RCT of a highly complex care intervention. We have shown that however committed trial teams are to delivering a standardised intervention across multiple sites, in reality, the intervention will be shaped and constrained by historical path dependencies and local realities.

### Clinical and practice considerations

In chronic heart failure, episodes of acute deterioration are common and if not promptly treated may lead to rapid decompensation. An assumption underlying SUPPORT-HF2 is that earlier detection of deterioration using the remote monitoring equipment (blood pressure, body weight, particular symptoms) could trigger rapid intervention that would prevent decompensation. But heart failure is a very complex condition with heterogeneous aetiology and frequent comorbidities, which means that many patients will be ‘exceptions’ to the standard algorithm and a high degree of personalisation of care is often required. For this reason, it will be hard to demonstrate in a trial that a standardised intervention significantly impacts on outcomes such as hospital admission rates or contacts with the health service.

Secondary care heart failure nurses emphasised safety concerns for the often very complex and unstable patients attending their clinics. Most took a strongly precautionary stance (possibly beyond that intended and expressed in the protocol’s exclusion criteria) that patients should not be recruited if there was a potential for harm. Such a restrictive recruitment strategy, whilst understandable from a professional perspective, not only slowed recruitment but may also have diluted the effect of the intervention (since patients likely to benefit from prompt efficient medication optimisation and enhanced monitoring rarely entered the trial at all). This conundrum is relevant to the design of any future trial of telehealth-supported care in heart failure.

### Strengths and limitations

This qualitative study drew on a large volume of data to understand variability in individual adoption and organisational assimilation of central remote specialist support in a telehealth trial for heart failure. A multidisciplinary research team was involved in the analysis of the data, combining an understanding of clinical aspects and current service models in heart failure, with expertise on the qualitative evaluation of digital health solutions and the social study of technology. Whilst we sought to draw on a maximum variation sample and interview participants with different experiences with the technology, those who had negative views may be under-represented. We were unable to identify patients who had declined to participate in the trial (although we did interview patients who withdrew). As trial participants, our interviewees were not necessarily representative of heart failure patients in general. Community HFSNs who had refused to refer patients to the trial were also unavailable for the interview by our team. The slow recruitment of participants in the trial was due partly to the non-engagement of front-line nurses, but the reasons for their resistance could only be explored indirectly.

## Conclusions

This paper has attempted, using socio-technical theory and the NASSS framework, to contextualise some of the SUPPORT-HF2 trial findings and explain the apparent lack of efficacy of a complex intervention involving both technology and human input. To summarise, in sites with insufficient specialist input to heart failure management in primary care, SUPPORT-HF2 was seen as a potentially useful component to patient care. At sites where care pathways in the community were relatively comprehensive, patient recruitment was highly selective and focused on patients who had had less to gain from the intervention. Our findings suggest, therefore, that the trial has not demonstrated definitively that the intervention is ‘ineffective’.

Complex socio-technical interventions, even when implemented in a so-called standardised way with uniform inclusion and exclusion criteria, are inevitably implemented differently in different local settings because of how individual staff members interpret the technology and the trial protocol and because of the practical realities and path dependencies of local organisations. Site-specific iteration and embedding of a new technology-supported complex intervention may be required (in addition to co-design of the user interface) before such interventions are ready for testing in clinical trials.

## Data Availability

The datasets used and/or analysed during the current study are available from the corresponding author on reasonable request.

## Appendix

### Additional data on the SUPPORT-HF2 trial

The SUPPORT-HF2 study hypothesis was: … in patients with heart failure, home monitoring with an integrated risk prediction and disease management service, which provided tailored alerts and advice to patients and clinical decision support to healthcare practitioners, is more effective in optimising medical therapy than home monitoring with the same monitoring equipment but without the use of the integrated data analysis and a centralised decision support service to advise general practitioners [7].

Adult patients with diagnosed heart failure were eligible for the SUPPORT-HF2 trial if they were assessed as having the potential to benefit from home monitoring and management (average self-assessed NYHA classes II to IV in the week before randomisation, or BNP > 100 pmol/L/NT-pro-BNP > 130 pmol/L within 30 days prior to randomisation, or at least two unmet treatment targets) and if they were at high risk of adverse outcomes (estimated one-year mortality risk > 10% or at least one hospital admission related to heart failure in the previous 12 months). Patients with heart failure with reduced ejection fraction (HFREF) and those with heart failure with preserved ejection fraction (HFPEF) were eligible. Optimal management of the former involves the use of up to 4 classes of drugs and in some cases device implantation, with symptomatic and prognostic benefit [35]. Optimal management of the latter hinges around diuretics for symptom control, but to date, there are no therapies with proven prognostic benefit [36]. Exclusion criteria included ‘any significant disease including critical, unstable or end-stage heart failure which, in the opinion of the investigator, may either put the participant at risk because of participation in the trial, or may influence the result of the trial, or the participant’s ability to participate in the trial’ ([8], p.56).

The CCM team in SUPPORT-HF2 was based in one of the sites (the ‘hub’) and supported 6 additional sites across the UK. As it would have been impossible to fully blind participants and clinicians to treatment options (home monitoring and home monitoring with remote specialist input), the study employed partial blinding, using an ‘attention control’ rather than a ‘usual care control’ arm. This meant participants were instead blinded to the study hypothesis so as to minimise potential ‘loser’ bias. To enable further comparison with usual care in the absence of facilitated self-monitoring, a third trial arm was established from patients who agreed to recruitment but were found ineligible in the run-in phase.

A total of 363 patients were assessed for eligibility, of whom 128 were excluded (96 declined; 22 did not meet criteria; 5 had other reasons; 5 died before screening). A total of 202 patients were randomised to the control or intervention arm. A further 25 of 33 patients who failed the run-in phase agreed to be followed up as part of the third trial arm to approximate ‘usual care’ [8].

The primary endpoint of SUPPORT-HF2 was used of recommended therapy defined as treatment consistent with the current guidelines for the management of patients with chronic heart failure. Additional predefined primary endpoint was improvement in patients’ physical well-being as assessed by Minnesota Living with Heart Failure Questionnaire and New York Heart Association (NYHA) scales and also by physiological measures including brain natriuretic peptide (BNP) level, heart rate and serum potassium in the ideal range. Secondary endpoints also included clinical safety as assessed by a composite of cardiovascular death, cardiovascular admissions (including renal failure and hypotensive episodes) and unscheduled outpatient visits.

## Abbreviations

CCM: Central clinical management
CI: Chief investigator
GP: General practitioner
HFSN: Heart failure specialist nurse
PI: Principal investigator
RCT: Randomised controlled trial
SCALS: Studies in Co-creating Assisted Living Technologies
SUPPORT-HF: Seamless User-centred Proactive Provision Of Risk-stratified Treatment for Heart Failure
